# Assessing the Value of DNA Barcodes and Other Priority Gene Regions for Molecular Phylogenetics of Lepidoptera

**DOI:** 10.1371/journal.pone.0010525

**Published:** 2010-05-07

**Authors:** John James Wilson

**Affiliations:** Department of Integrative Biology, University of Guelph, Guelph, Ontario, Canada; Landcare Research, New Zealand

## Abstract

**Background:**

Despite apparently abundant amounts of observable variation and species diversity, the order Lepidoptera exhibits a morphological homogeneity that has provided only a limited number of taxonomic characters and led to widespread use of nucleotides for inferring relationships. This study aims to characterize and develop methods to quantify the value of priority gene regions designated for Lepidoptera molecular systematics. In particular, I assess how the DNA barcode segment of the mitochondrial COI gene performs across a broad temporal range given its number one position of priority, most sequenced status, and the conflicting opinions on its phylogenetic performance.

**Methodology/Principal Findings:**

Gene regions commonly sequenced for Lepidoptera phylogenetics were scored using multiple measures across three categories: practicality, which includes universality of primers and sequence quality; phylogenetic utility; and phylogenetic signal. I found that alternative measures within a category often appeared correlated, but high scores in one category did not necessarily translate into high scores in another. The DNA barcode was easier to sequence than other genes, and had high scores for utility but low signal above the genus level.

**Conclusions/Significance:**

Given limited financial resources and time constraints, careful selection of gene regions for molecular phylogenetics is crucial to avoid wasted effort producing partially informative data. This study introduces an approach to assessing the value of gene regions prior to the initiation of new studies and presents empirical results to help guide future selections.

## Introduction

The Lepidoptera are a globally distributed, charismatic group which has seen extensive taxonomic attention yet still can be considered ‘unknown’. Current estimates for the global total of lepidopteran species range from 280, 000 to 1.4 million species [1] but only 100, 000 have been described [2], representing a critical gap in our knowledge. Additionally, higher taxonomic relationships within the most species rich group (containing 98% of all known species) – Ditrysia - are still ‘shrouded in mystery’ [3].

Despite apparently abundant amounts of observable variation and species diversity, the order exhibits a morphological homogeneity that has provided only a limited number of taxonomic characters and led to widespread use of DNA sequences for inferring relationships (e.g. [4], [5], [6]). DNA sequence databases are growing at an exponential rate [7] but continue to exhibit uneven taxonomic distributions. Many genes are available for a limited set of exemplar taxa but only one or two genes are available for the majority of species (see [8], [9]). Therefore it is not surprising that the value of taxon and character sampling in phylogenetic datamatrices continues to be fiercely debated in the literature [10]. The debate is particularly relevant to those studying Lepidoptera [11] as two big science projects, ATOL (http://www.leptree.net) and DNA barcoding (http://www.lepbarcoding.org), alternatively promote increased genomic or taxon coverage respectively.

DNA barcoding refers to the technique of sequencing a short fragment of the mitochondrial *cytochrome c oxidase subunit I* (COI) gene from a taxonomically unknown specimen and performing comparisons with a reference library of sequences of known species origin to establish a species-level identification. The technique has gained acceptance among the taxonomic community but the use of the barcode fragment in phylogenetics, especially without additional genetic data remains controversial [12].

These two big science projects are however largely complementary [13] and knowledge of the Lepidoptera phylogeny should benefit from a strong community movement to standardize molecular sequencing efforts (http://www.lepsys.eu) and avoid the Tower of Babel of molecular systematics [3], [14]. The LEPSYS.eu consortium is promoting the use of priority molecular markers for phylogenetic studies, with the goal of emulating the successes of plant systematists and their extraordinary database of homologous sequences from thousands of plant species. COI, from which the DNA barcode is derived [15], [16] and the nuclear gene *elongation factor-1 alpha* (EF1a), have been sequenced most extensively for Lepidoptera and are recommended by the consortium as the first gene regions to sequence in any new study.

While the designation of priority gene regions is certainly commendable, the presence of advantageous characteristics for phylogenetic analysis in these genes has been questioned [5], [17], and the temporal ranges (i.e. taxonomic levels) over which different gene regions are most informative have never been thoroughly investigated in broad comparisons across the order. Many authors assess the phylogenetic value of datamatrices, often ambiguously termed utility, through an *ad hoc* combination of the number of potentially informative characters and the quality, ‘accuracy’ and support of an inferred phylogeny (e.g. [18]). Value is often measured in relative terms; morphological versus molecular data [19], this gene versus that gene [20]. For example, Nazari et al. [21] looked at relationships within Parnassiinae (Papilionidae) and found conflicting, weak results from mtDNA compared to nuclear and morphological data, and that nuclear genes were particularly good at resolving deeper nodes. In contrast, Warren et al. [6] looked at relationships within Hesperiidae and found good support from COI and EF1a, but conflicting results from another nuclear gene, *wingless* (WG).

Consequently, the objective of this study is to characterize and develop methods to quantify the value of priority gene regions designated for Lepidoptera molecular systematics. In particular, I will assess how the DNA barcode segment of COI performs across a broad temporal range given its number one position of priority, most sequenced status, and the conflicting opinions on its phylogenetic performance [21] [Bibr pone.0010525-Sperling1]–[23].

To undertake these goals, it is important to develop objective measures by which gene regions can be judged. A useful guide could be the criteria used recently to select the plant DNA barcode [24] although different approaches have been undertaken (e.g. [25]) to target the common problem addressed in this study. The Plant Working Group followed the Consortium for the Barcode of Life's data standards and guidelines for locus selection (http://www.barcoding.si.edu/protocols.html) with three specific categories included. Modified slightly for systematics above the species-level the categories are:

### Practicality

This encompasses: a) universality- which loci can be routinely sequenced across Lepidoptera; and b) sequence quality- which loci are most amenable to the production of bidirectional sequences with few or no ambiguous base calls?

### Phylogenetic utility

Wortley and Scotland [19] delineate this term as intrinsic properties of a datamatrix measured prior to phylogenetic analysis. Measures include the character-taxon ratio, the number of variable or parsimony informative characters and phenetic distances between taxa (Table 1). Cameron and Whiting [25] also used ‘utility’ in the context of the number of variable characters of various classes.

**Table 1:** Measures of phylogenetic utility and signal used in this study.

Measure		Notes
Phylogenetic utility	*A*	Number of aligned characters; equivalent to number of columns in an aligned matrix.
	*V*	Number of variable characters; *A* excluding invariant characters.
	*PI*	Number of parsimony-informative characters; *V* excluding autapomorphies.
	*M*	Minimum number of character-state changes.
	*G^t^*	Number of terminals (species) in datamatrix.
	Character-taxon ratio	*A*/*G^t^*
	p	Phenetic distance between taxa, averaged for all pairwise comparisons.
Phylogenetic signal	*S*	Tree length; minimum number of state changes on the cladogram in question.
	*CI*	Ensemble consistency index; *M*/*S*
	*G*	Greatest number of character state changes on any cladogram.
	*RI*	Ensemble retention index; (*G*-*S*)/(*G*-*M*)
	*M^t^*	Number of taxa included in the test (e.g. number of families).
	*PMT*	Proportion of monophyletic taxa; Number of monophyletic taxa/*M^t^*
	*S^t^*	Minimum number of clades a taxon exhibits on cladogram in question; summed for all test taxa.
	*TCI*	Taxon consistency index; *M^t^*/*S^t^*
	*TRI*	Taxon retention index; (*G^t^*-*S^t^*)/(*G^t^*-*M^t^*)

### Phylogenetic signal

This category can be interpreted as the ability of a datamatrix to group taxonomically related taxa together [26] or ‘accuracy’ of a phylogenetic hypothesis. Although the accuracy of phylogenetic inference can never be known [27], except when using simulated evolution (e.g. [28]), proxy measures are commonly used. Signal is necessarily measured after phylogenetic analysis and can be measured a) through character congruence within the current datamatrix quantified by the consistency and retention indices ([29], [30], Table 1) or; b) through congruence of the hypothesis with an inference produced from independent sources of data (taxonomic congruence). As the current classification represents a consensus phylogenetic hypothesis, measures of phylogenetic signal can be formalized through the designation of concordance groups derived from taxonomy (e.g. [11], [27], [31], but see [32]). Although taxonomic congruence is typically assessed qualitatively [30], in this study I present quantitative measures adapted from the character consistency and retention indices used to assess character congruence ([29], Table 1).

## Results and Discussion

### Practicality

**Figure 1:**
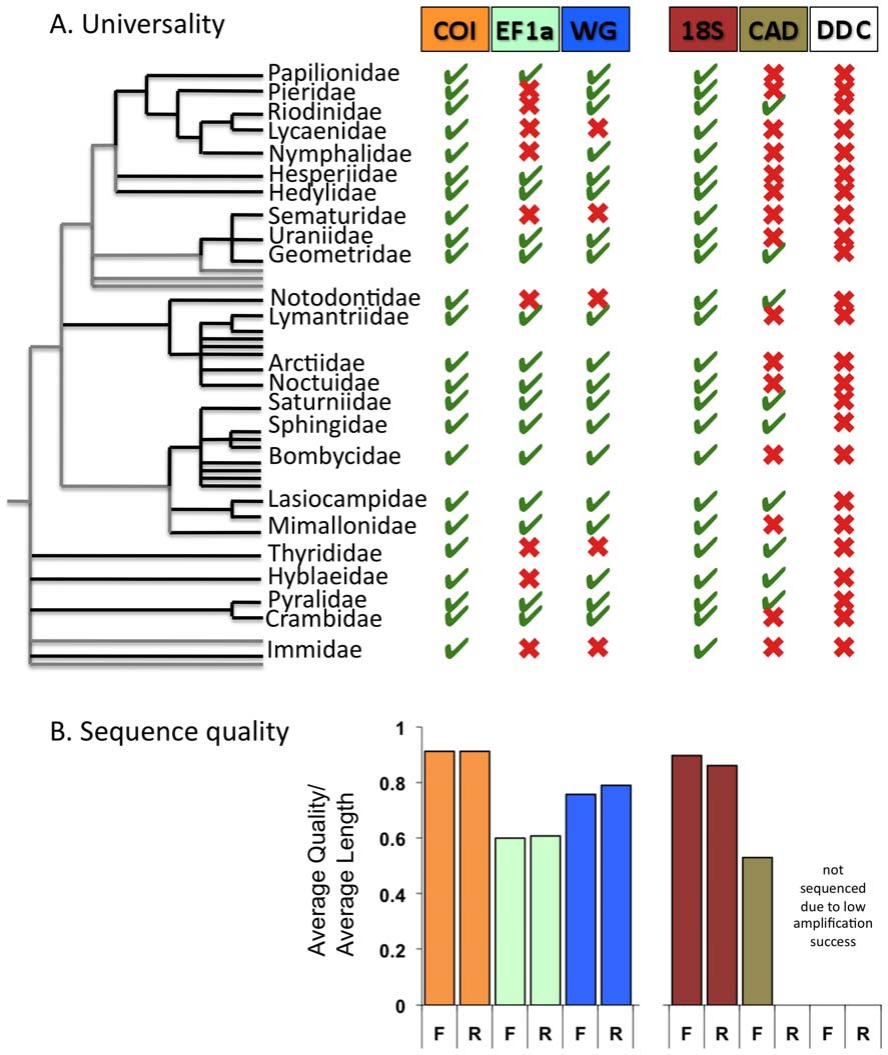
Results of the experiment to test the practicality of sequencing six of the commonly sequenced gene regions for Lepidoptera molecular systematics. A). Universality of primers for the tested gene regions against the taxonomic scheme of Pogue [50]. Families included in the test dataset are named on branches of the tree, unnamed branches are families for which no specimens were available. Families within a superfamily are connected by black line. A tick indicates a distinct band was present on the E-gel for at least one specimen of the family, an X indicates no bands were visible. B). Sequence quality was measured in CodonCode Aligner using Phred algorithm. F refers to the forward sequences and R refers to the reverse.

The first set of experiments consisted of determining the practicality of obtaining sequences of gene regions commonly employed for Lepidoptera phylogenetics with standard high-throughput molecular sequencing techniques across a broad taxonomic sample (Figure 1). It is common for research groups to use a single recipe for PCR cocktails and single thermocycling profile for all primer combinations and gene regions sequenced in their labs (see http://nymphalidae.utu.fi/Nymphalidae/Molecular.htm). I modeled techniques commonly used in molecular systematic labs in order to reproduce what any lab attempting to sequence new genes would try first (e.g. [34]). The practicality category encompassed scoring primer universality and sequence quality. Not surprisingly, primers for the multi-copy gene regions, COI and 18S rDNA, produced the most distinct bands on the gels (100%), indicating successful PCR amplification (Figure 1A). Although taxon selection was limited to a small number of species, all primers, except DDC, appear to have a broad taxonomic range with bands for both macrolepidopteran and microlepidopteran families and no clear taxonomic pattern to amplification failures. The CAD and EF1a primers seemed particularly poor at amplifying product in butterflies (the superfamily at the top of the tree in Figure 1A), a somewhat surprising result since much effort has been focused on collecting molecular data in this group. Failures in EF1a and WG seemed to match taxonomically; 15 families with distinct bands for EF1a also produced distinct bands for WG, although 4 additional families were amplified for WG. Since at least one gene was successfully amplified and sequenced from every specimen, it seems unlikely there were problems with DNA quality. It does seem quite possible that a second round of optimization of reaction and thermocycle conditions could produce bands for the missing regions. For example, for those regions that amplified poorly, it is likely that MgCl2 concentrations were not optimal [34]. Also it is usual activity in a molecular phylogenetics project to re-design and optimize primers after an initial test run. However, these were not tested in this study, as I was specifically interested in identifying gene regions that were successfully amplified under standard conditions for high-throughput processing with minimal optimization. The results for sequence quality matched closely with the results for primer universality. COI and 18S were the highest quality sequences (0.91 and 0.88 respectively), WG was intermediate (0.78) between these and EF1a (0.61) and CAD (0.53) sequences (Figure 1B). That the COI sequences were the highest quality was not surprising given the historical efforts undertaken to optimize primers and protocols for this gene (e.g. [35]).

The region rankings for primer universality and sequence quality seen in this study closely resembled the priority gene ranking of the LEPSYS.eu consortium. Two exceptions were 18S and EF1a. The 18S gene has not been selected as a priority gene region even though it proved easy to produce high quality sequences in all taxa. This is most likely because of problems establishing primary homology in length variable regions [36]. There were fewer EF1a amplifications than WG amplifications, despite the former's position as number two on the priority ranking. This may be an effect of the primer pair chosen for EF1a. While COI and WG have only a limited number of primer options available from published studies, numerous different regions have been used to amplify EF1a fragments (e.g. [37]), and I could have inadvertently chosen a set that was not optimal for my taxon sample. The problem with a plethora of competing primer options could also be relevant to CAD. Because different research groups are sequencing different, and often non-overlapping fragments of the same genes, this limits inclusion of the genes in composite supermatrix analyses (see below). Surprisingly, this was also a problem seen with COI when many species had to be excluded from the utility and signal experiments, because the fragment of COI available on GenBank did not overlap with the DNA barcode region. This demonstrates that it might be equally as important for the LEPSYS.eu consortium to specify a more precise fragment and universal primers alongside designations of standard gene regions.

### Phylogenetic utility

A search of GenBank, combined with new sequences produced in this study, recovered 977 species from macrolepidopteran families and potential microlepidopteran sister families with sequences available for all the following three gene regions: COI (barcode fragment), Ef1a and WG. Sequences were downloaded and aligned, and a datamatrix was created for each gene. From these matrices I estimated, using PAUP, standard measures of utility, defined for the purpose of this study as properties of the matrices measured prior to analysis [19]. The other gene regions included in the practicality experiments (18S, CAD, DDC) were not included in the utility and signal analyses due to the relatively low number of sequences available on GenBank and lack of overlap of species sequenced for theses genes and species sequenced for the three most common genes COI, EF1a and WG. Including 18S or CAD would have led to datasets which were not comparable. There are actually very few Lepidoptera genera with 18S sequences from multiple species on GenBank, due to the fact this gene is often sequenced for investigations into deeper taxonomic levels. The same is true for CAD, where sequences exists they are not easily aligned, often not homologous fragments and not available for multiple species from within a genus.

The simplest measure of utility is simply the number of columns in the aligned matrix (A). EF1a had the highest score for A at 1006. Trimming the sequences downloaded from GenBank was especially difficult for EF1a as no standard region is amplified and sequenced across research groups. Obtaining a maximal score necessitated lots of missing data, coded as Ns, and produced the only datamatrix with no overlap of non-ambiguous data between some taxa. This missing data could be exerting an effect on the utility scores. COI had the intermediate score for A, after being trimmed to the DNA barcode region. Many species had to be excluded from the analysis because the COI sequence on GenBank did not overlap with the DNA barcode fragment. As the same species were included in each datamatrix, the character-taxon ratio was directly proportional to measures of A. It will always be highly dependent on A, in which case WG would often have the lowest score. Gene number is closely associated with A and is another factor often highly regarded as an indicator of utility. Gene number is often reported in the title of papers [5], [13, [21] and the assumed value of gene number as a measure of utility may be an artefact of using bootstrap support to evaluate phylogenetic hypotheses (e.g. [34]). Bootstrap values increase as a function of A regardless of the quality of the phylogeny.

**Figure 2:**
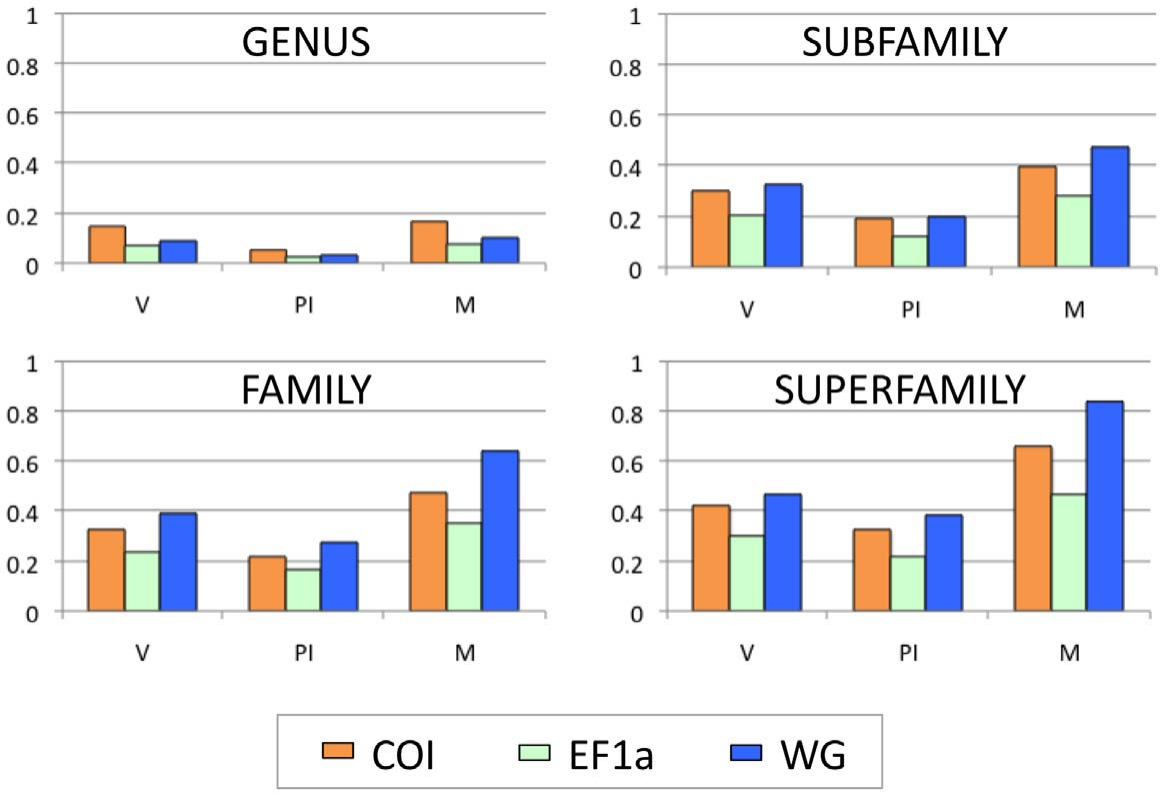
Phylogenetic utility scores. On the y-axes, the proportion refers to A, the aligned sequence length; A was 658 for COI, 1006 for EF1a and 409 for WG. V is the proportion of variable characters, PI is the proportion of parsimony informative characters and M is the proportion of the minimum number of character state changes (see Table 1).

The number of variable (V) or parsimony-informative characters (PI) and minimum number of state changes (M) are properties of A which may be more informative measures of utility. I found that all these measures were closely correlated to one another (Figure 2), but did not relate to A. Despite having the lowest score for A, WG had the highest PI score (measured as a proportion of A; Figure 2) and scored the highest in all other measures of phylogenetic utility at all taxonomic levels above genus. COI scored highest for V, PI and M within genera. The utility scores showed similar relationships between the gene regions at all taxonomic levels, and all increased at deeper taxonomic levels where more species, and consequently more opportunity for variable characters, were included in each calculation (Figure 2). Wahlberg and Wheat [34] noted the new nuclear genes they investigated in their study had similar levels of parsimony-informative sites between 30–50% of all sequenced sites, which is similar to the values presented here (Figure 2). Despite the large differences in A, the absolute number of parsimony-informative characters for each gene region was remarkably similar across all datamatrices ranging from 309 to 472. It is worth noting that homoplasy, often cited as an indicator of utility, can only be inferred from a cladogram, and never known for certain, thus it is useless as a measure for determining utility prior to phylogenetic analysis [19] and why I consider it as a measure of signal for the purposes of this study.

In contrast to Wortley and Scotland [19] I found that all measures of phylogenetic utility were roughly correlated. The only inconsistent measures were A and consequently the character/taxon ratio. A is the only measure not dependent on the taxon sample, and perhaps our different findings can be explained by the fact that their study included datamatrices containing different numbers of taxa sampled across different taxonomic levels, whereas, measures were structured by taxonomic level and averaged across a large range of genetic divergences in this broad lepidopteran sample.

### Phylogenetic signal

**Figure 3:**
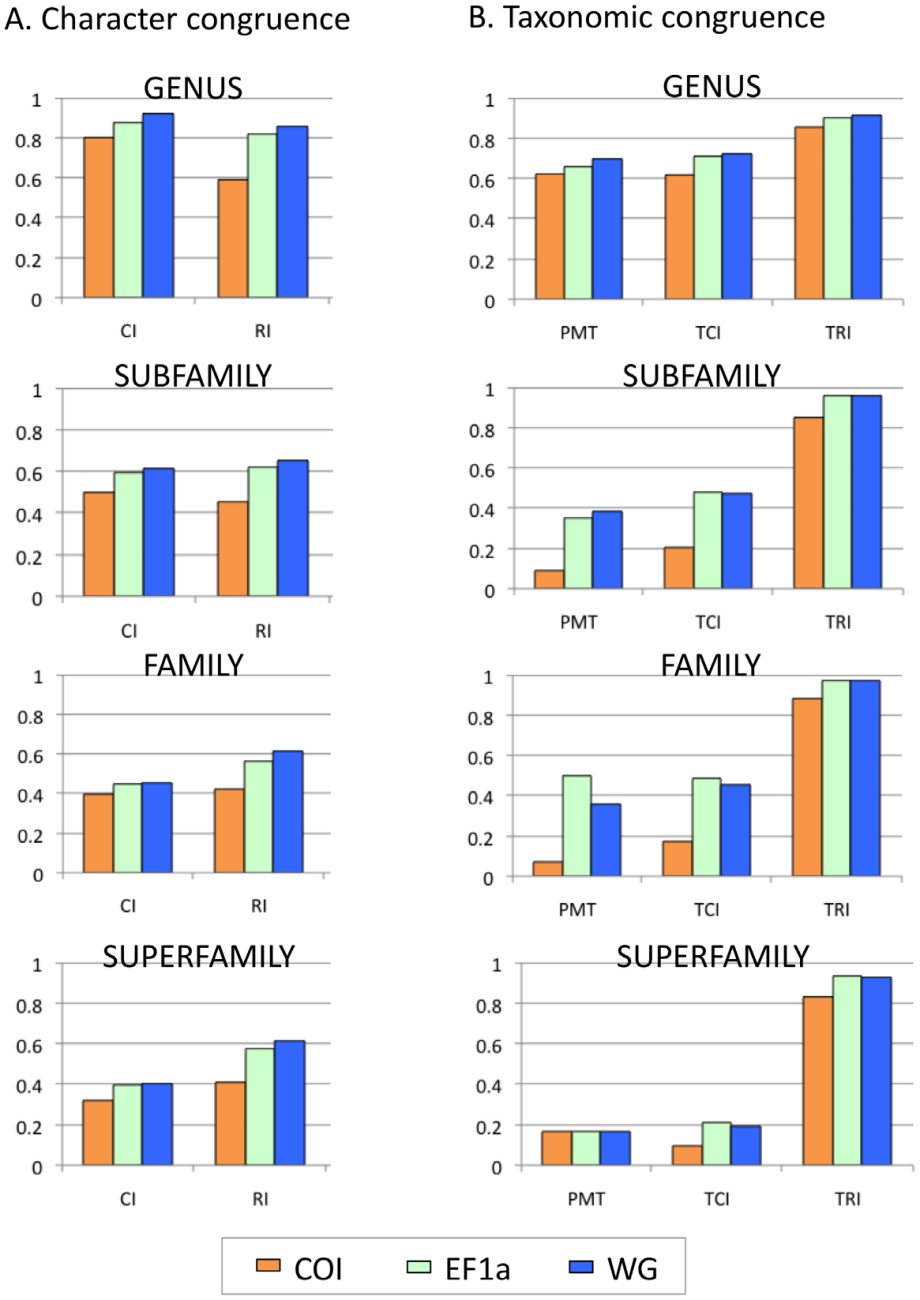
Phylogenetic signal scores. A). Character congruence. Note that lower values of CI and RI indicate more homoplasy in the datamatrix. B). Taxonomic congruence. All abbreviations refer to Table 1; PMT is the proportion of monophyletic taxa, TCI is the taxon consistency index and TRI is the taxon retention index.

All three genes included in this study have been previously promoted as having strong phylogenetic signal [6], [21], although previous assessments have been largely qualitative and *ad hoc*. Phylogenetic signal can be defined as the ability of a datamatrix to group taxonomically related taxa together and can be quantified through character congruence (within the dataset) or taxonomic congruence (between datasets) (Table 1). Character congruence measured across large datamatrices through the consistency index (CI) is perhaps not very informative (Figure 3) because homoplasy is almost guaranteed to be present, given the limited number of possible nucleotide substitutions and the historical divergence times. The retention index, which corrects for the number of taxa is likely to be more informative and showed that WG had the strongest signal, EF1a the intermediate, and COI the weakest signal across all taxonomic levels (Figure 3). Signal measured through character congruence decreased in all genes from genus to subfamily (Figure 3). However, there are conflicting opinions about the impact of the level of inferred homoplasy on phylogenetic signal [38]. Character congruence seemed closely correlated with taxonomic congruence in this study, providing some justification for the inclusion of character congruence as a measure of signal (Figure 3). This may especially be the case where taxonomic congruence measures are not applicable because no ‘known’ phylogeny exists or there is no logical means of partitioning ‘independent’ data sources (see [29] for a review of this debate).

Taxonomic congruence, typically assessed qualitatively by systematists, was assessed quantitatively in this study through three measures: (1) the proportion of monophyletic taxa, (2) the ensemble taxon consistency index and (3) the ensemble taxon retention index (see Table 1). All three measures appeared highly correlated, although with a larger number of taxa where monophyly is probabilistically least expected, the TCI and TRI may represent more informative measures. The TCI and TRI may also be less sensitive to error due to the arbitrary nature of taxonomic ranks and the fact that many of the taxa included may not represent natural groups. As judged by the three measures, the signal in all genes was very similar at the genus level (TCI ranged from 0.62–0.72; Figure 3). Moving up the taxonomic hierarchy to subfamily, EF1a and WG have reasonable signal and the values are fairly similar (0.47), but signal in COI was only about half as strong (0.20) based on the TCI values. Family results were similar to subfamily but at the superfamily level low signal was observed for all three genes. The prevailing view of low signal in COI at deep divergences was supported by this study, however, at shallower divergences (genus level) COI signal was comparable with the nuclear genes.

### Concluding remarks

The literature regarding the use of molecular sequence data in phylogenetic inference has often relied upon model-based or qualitative measures of utility, a term which itself has been used ambiguously. However, it is crucial to have reliable empirical results when making recommendations about which gene regions to sequence large-scale as standards [24]. In this study I developed objective measures for assessment of fundamental qualities pertinent to the assembly of a molecular datamatrix. These measures in three categories; practicality, phylogenetic utility and phylogenetic signal, were then applied to single-gene datamatrices, each containing 977 species of Lepidoptera. The categories and measures used in this study have not focused on model-based properties of the data, for example, the function of the genes and associated modes of molecular evolution. As a result of this distinction, these criteria are also applicable to other types of phylogenetic characters (e.g. morphology), with minor modifications to the practicality component. This could be in the form of a measure of the ease of scoring morphological characters by non-specialists. While molecular evolution is undoubtedly an interesting avenue of research, incorporating process-models into phylogenetic hypothesis testing, involves additional assumptions which are always likely to be arbitrary, over simplified, or even just plain wrong [33], [39], [40].

I found that alternative measures within a category were often highly correlated, but that high scores across one category did not necessarily translate into high scores across another. The DNA barcode fragment of COI was easier to sequence than the other genes, and had high scores for utility but low signal above the genus level. COI's number one position of priority in the LEPSYS.eu list could be justified due to the ability to confirm the species identity of a new specimen being sequenced [13]. This is especially important given the prevalence of misidentified sequences/specimens currently being submitted to GenBank.

Whole genome phylogenetics (phylogenomics [34]) has been considered prohibitively expensive but is becoming increasingly feasible. For example, mitochondrial genomics based phylogenomics can be done for less than $500 a genome and will become more mainstream as sequencing costs decrease over the next few decades. Methodological advances are required to effectively analyze such large amounts of data. Most recently published phylogenetic hypotheses are reconstructed from datamatrices containing few genes, and sometimes only one [41]. A single short gene fragment may well be sufficient depending on the phylogenetic question under investigation. However, given limited financial resources and time constraints, careful selection of target gene regions can be crucial to avoid wasted effort leading to the production of sub-informative data. This study introduces an approach to assessing the value of gene regions prior to the initiation of new studies and presents empirical results to help guide future selections.

## Materials and Methods

### Practicality

Seventy-two species of Lepidoptera were selected from 60, 000 specimens collected in Area de Conservacion Guanacaste, Costa Rica and shipped to the Canadian Centre for DNA Barcoding (CCDB) as part of “BioLep Project” (http://www.bolinfonet.org/casestudy/index.php/display/study/20) [42]. The selection included at least one species from each macrolepidopteran family available, plus species from potential outgroup microlepidopteran families (Table S1). DNA was extracted from legs using Qiagen DNAeasy Kit following the manufacturers instructions for animal tissue (www.quiagen.com). Primer pairs expected to amplify product of approximately 500 bp, were obtained for COI, EF1a, WG, 18S rDNA (18S), Carbamoyl phosphate synthase II, Aspartate carbamoyltransferase, Dihydroorotase (CAD) and dopa decarboxylase (aromatic L-amino acid decarboxylase) (DDC) and used for PCR in standard protocols. High-throughput PCR set-up followed http://www.dnabarcoding.ca while thermocycling profiles followed http://nymphalidae.utu.fi/Nymphalidae/Molecular.htm.

All primers were tailed with M13 except for LepF1 and LepR1 (Table 2). Universality success was scored based on the presence of a distinct band on an E-gel [43]. PCR products were sequenced using M13 primers in standard protocols (http://www.dnabarcoding.ca) with the exception of COI, which was sequenced using the PCR primers. Chromatograms were imported into CodonCode Aligner (www.codoncode.com) and summarized scores of sequence quality were generated from raw files.

**Table 2:** List of primers used in this study.

Primer name	Sequence (5′>3′)	Gene	Reference
LepF1	ATTCAACCAATCATAAAGATATTGG	COI	[51]
LepR1	TAAACTTCTGGATGTCCAAAAAATCA	COI	[51]
Cho (E234F)	GTCACCATCATYGACGC	EF1a	[52]
Juke (E600rc)	CTCCTTACGCTCAACATTC	EF1a	[52]
LepWG1	GARTGYAARTGYCAYGGYATGTCTGG	WG	[53]
LepWG2a	ACTICGCARCACCARTGGAATGTRCA	WG	[53]
rc18H	GCTGAAACTTAAAGGAATTGACGGAAGGGCAC	18S rDNA	[54]
18L	CACCTACGGAAACCTTGTTACGACTT	18S rDNA	[54]
CAD743nF	GGNGTNACNACNGCNTGYTTYGARCC	CAD	[34]
CAD1028R	TTRTTNGGNARYTGNCCNCCCAT	CAD	[34]
DDC3.2sF	TGGYTICAYGTIGAYGCNGCNTAYGC	DDC	[34]
DDCdegR3	CCCATNGTNACYTCYTC	DDC	[34]
M13F(-21)	TGTAAAACGACGGCCAGT		[55]
M13R(-27)	CAGGAAACAGCTATGAC		[55]

### Phylogenetic utility

I mined GenBank for macrolepidopteran species, and species from potential microlepidopteran sister families, with sequences available for all three gene regions: COI (barcode fragment), EF1a and WG. The dataset was supplemented with newly generated sequences from the practicality experiment above, available at www.barcodinglife.org (Published Project LGC). Sequences from species meeting these criteria were downloaded creating three datamatrices with 977 species (Table S2). Sequences were trimmed and aligned in BIOEDIT [44] using CLUSTALW and with minor modifications by eye. Measures of phylogenetic utility (Table 1) were calculated in PAUP [45]. Values were measured within taxa for those represented by three or more species in the datasets (Table 3) and averaged for four taxonomic levels: Genus, Subfamily, Family and Superfamily.

**Table 3:** The taxonomic structure of datamatrices used to measure phylogenetic utility and signal.

Taxonomic rank	# of taxa	# of concordance groups i.e. taxa containing >1 species	# of taxa containing >2 species
Species	977	n/a	n/a
Genus	200	109	56
Subfamily	53	34	27
Family	20	13	13
Superfamily	11	6	6

Taxon membership followed LepIndex (www.nhm.ac.uk/research-curation/projects/lepindex/) or NCBI taxonomy database (http://www.ncbi.nlm.nih.gov/Taxonomy/).

### Phylogenetic signal

Aligned datamatrices were analysed using the phenomenological method of maximum parsimony in TNT (new technology searches using the default section and ratchet options) [46]. Genus, subfamily, family and superfamily groups were designated as concordance groups (see [27], [31], [47], [48], [49]) for tests of phylogenetic signal through taxonomic congruence (Table 3). Quantification was incorporated in the form of three measures: (1) the proportion of monophyletic taxa, (2) the ensemble taxon consistency index and (3) the ensemble taxon retention index - modeled after the character consistency and retention indices used in cladistics (see [29], Table 1). Values for these indices were obtained by constructing datamatrices of characters relating to group membership (i.e. 1 = member, 0 = non-member) and scoring these characters in PAUP on the trees produced from the parsimony analysis of the molecular characters. The best possible score is 1 and higher values indicate the taxa are closer to monophyly. Character congruence was measured through the consistency and retention index. Values were measured within taxa for those represented by three or more species in the datasets (Table 3) and averaged for four taxonomic levels: Genus, Subfamily, Family and Superfamily.

## Supporting Information

Table S1 Specimens used in practicality experiment.
(0.03 MB XLS)

Table S2 Sequences used in phylogenetic utility and phylogenetic signal experiments.
(0.18 MB XLS)
